# Supplementary material for: The causal role of circulating inflammatory markers in osteoporosis: a bidirectional Mendelian randomized study
Source: Front Immunol. 2024 Jul 18;15:1412298. doi: 10.3389/fimmu.2024.1412298 (PMC11291241; doi:10.3389/fimmu.2024.1412298)
Supplement: Supplementary file 1 [file DataSheet_1.zip › supplementary material/OSTEOPOROSIS-CIM/GCST90274810.tsv.gz_leave_one_out.pdf]

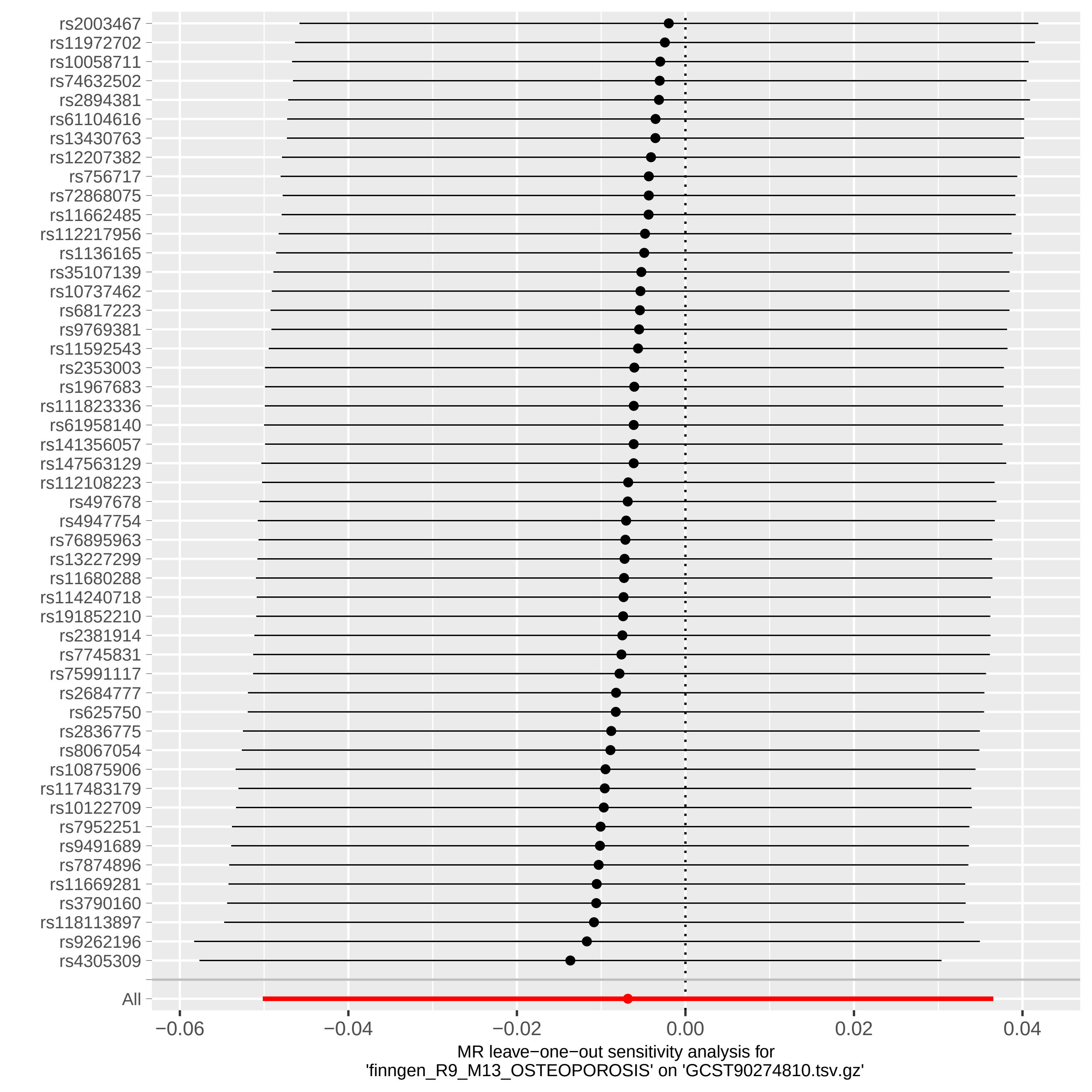

rs2003467
rs11972702
rs10058711
rs74632502
rs2894381
rs61104616
rs13430763
rs12207382
rs756717
rs72868075
rs11662485
rs112217956
rs1136165
rs35107139
rs10737462
rs6817223
rs9769381
rs11592543
rs2353003
rs1967683
rs111823336
rs61958140
rs141356057
rs147563129
rs112108223
rs497678
rs4947754
rs76895963
rs13227299
rs11680288
rs114240718
rs191852210
rs2381914
rs7745831
rs75991117
rs2684777
rs625750
rs2836775
rs8067054
rs10875906
rs117483179
rs10122709
rs7952251
rs9491689
rs7874896
rs11669281
rs3790160
rs118113897
rs9262196
rs4305309
All
−0.06
−0.04
−0.02
0.00
0.02
0.04
MR leave−one−out sensitivity analysis for
'finngen_R9_M13_OSTEOPOROSIS' on 'GCST90274810.tsv.gz'
